# Neurocognitive Function and Quality of Life Outcomes in the ONTRAC Study for Skin Cancer Chemoprevention by Nicotinamide

**DOI:** 10.3390/geriatrics4010031

**Published:** 2019-03-25

**Authors:** Andrew J. Martin, Haryana M. Dhillon, Janette L. Vardy, Robyn A. Dalziell, Bonita Choy, Pablo Fernández-Peñas, Ann Dixon, Corrinne Renton, Gayathri St George, Niranthari Chinniah, Gary M. Halliday, Diona L. Damian, Andrew C. Chen

**Affiliations:** 1 NHMRC Clinical Trials Centre, University of Sydney, Sydney, NSW 2006, Australia; andrew.martin@ctc.usyd.edu.au; 2 Centre for Medical Psychology & Evidence-Based Decision-Making, University of Sydney, Sydney, NSW 2006, Australia; haryana.dhillon@sydney.edu.au (H.M.D.); Janette.vardy@sydney.edu.au (J.L.V.); Ann.dixon@sydney.edu.au (A.D.); corrinne.renton@sydney.edu.au (C.R.); 3 Sydney Medical School, Concord Clinical School, University of Sydney, Sydney, NSW 2006, Australia; 4 Concord Repatriation General Hospital, Sydney Concord, NSW 2139, Australia; 5 Dermatology and Bosch Institute, University of Sydney at Royal Prince Alfred Hospital, Camperdown, NSW 2050, Australia; robyn.dalziell@sydney.edu.au (R.A.D.); gary.halliday@sydney.edu.au (G.M.H.); andrewchen78@gmail.com (A.C.C.); 6 Dermatology, University of Sydney at Westmead Hospital, Westmead, NSW 2145, Australia; bonitachoy@hotmail.com (B.C.); pablo.fernandezpenas@sydney.edu.au (P.F.-P.); Gaya.stgeorge@sydney.edu.au (G.S.G.); Nirachinniah@gmail.com (N.C.); 7 Melanoma Institute Australia, North Sydney, Wollstonecraft, NSW 2065, Australia

**Keywords:** vitamin B3, cognitive aging, prevention

## Abstract

Nicotinamide (vitamin B3) has photoprotective effects and reduces skin cancer incidence in high risk patients. Nicotinamide also improves cognition in animal models. As part of the ONTRAC (Oral Nicotinamide To Reduce Actinic Cancer) phase III placebo-controlled, randomized trial to assess nicotinamide’s efficacy in skin cancer prevention, we included clinical neurocognitive function and patient-reported quality of life assessments at baseline and after 12 months of intervention in individuals with previous skin cancer in order to assess any effect of oral nicotinamide (500 mg po twice daily) on cognitive function and quality of life. In our sample of 310 participants who completed neurocognitive function testing at baseline and at 12 months, we were not able to detect any significant effect of oral nicotinamide on cognitive function nor on quality of life. Further studies of nicotinamide’s effects on cognition in humans might include individuals with pre-existing mild cognitive impairment, and it may be that higher doses of nicotinamide are required to significantly influence cognitive function compared to doses required to reduce skin cancer.

## 1. Introduction

As a precursor of nicotinamide adenine dinucleotide (NAD^+^), nicotinamide (vitamin B3) plays a central role in cellular energy metabolism. In the skin, nicotinamide is able to replenish cellular energy after ultraviolet radiation exposure [1], thereby enhancing the highly energy-dependent process of epidermal DNA repair [2]. Nicotinamide additionally has immune protective [3] and anti-inflammatory effects [4] which suggest its potential for skin cancer chemoprevention. Our multicenter phase III double-blind, randomised placebo-controlled trial (ONTRAC; Oral Nicotinamide to Reduce Actinic Cancer) found that oral nicotinamide (vitamin B3) significantly reduced the incidence of nonmelanoma skin cancer (basal cell and squamous cell carcinoma) by 23% relative to a placebo in a high risk population [5] (Australian New Zealand Clinical Trials Registry number ACTRN12612000625875). 

Secondary pre-specified objectives of ONTRAC included an evaluation of the effect of oral nicotinamide on neurocognitive function (NCF) and quality of life (QoL). The NCF objective was pursued given the evidence for the role of nicotinamide in cognitive and neurological function available at the time ONTRAC was designed. In mice, exposure to nicotinamide has been associated with improved short term memory [6], reduced cognitive decline in an Alzheimer’s model [6], and improved recovery from cortical contusion injury [7]. Nicotinamide has also been used as a neuroprotective agent in mouse models of stroke [7] where it is thought to act by increasing intracellular energy (adenosine triphosphate) [8]. In humans, lower dietary nicotinamide has been associated with increased dementia risk [9], and dementia is a known symptom of nicotinamide deficiency (pellagra) [10]. 

## 2. Results

Of the 386 patients randomized to ONTRAC, 310 and 290 took part in the NCF and QoL substudies respectively (Supplementary Figures S1 and S2). Substudy participants had comparable characteristics to the entire ONTRAC population (Comparison for NCF substudy shown in Table 1). The mean age of participants in the NCF substudy was 66 years (range 30–91); 37% were female. The mean baseline and change from baseline to month 12 scores are shown in Table 2 along with the estimated treatment effect from the linear model.

There were no differences between the arms in any neuropsychological test at baseline. All mean scores were within the expected range with the exception of verbal memory and learning in which both groups scored 1SD below the expected mean. There was no difference between the arms in the change score from baseline to 12 months, with the exception of the Digit span assessment which yielded a p-value below 0.05; however, this result remained consistent with the play of chance when taking into account the multiple comparisons performed. There was no difference between the arms in cognitive symptoms or in any of the QoL domains at any time point. 

## 3. Discussion

In our cohort of largely elderly, community dwelling skin cancer patients assessed over a 12-month intervention period, there was no compelling statistical evidence of an effect of nicotinamide on neurocognitive performance nor on QoL. The narrow width of the confidence intervals helps rule out the plausibility of nicotinamide causing a clinically important reduction in NCF or QoL. The effects of nicotinamide on NCF in individuals at higher risk of progression to dementia, for example those with early cognitive impairment but not frank dementia, is currently unknown. There are, as of yet, no published studies of nicotinamide use in this setting, nor as a preventive agent in individuals without cognitive deficit. Whilst some patients with Alzheimer’s Disease given nicotinamide adenine dinucleotide (NADH; 10mg daily) did show improvement in mini mental state examination and global deterioration scores [11], others did not [12]. Nicotinamide derivatives such as nicotinamide mononucleotide, nicotinamide riboside [13] and nicotinamide loaded lipid nanoparticles [14] have, at extremely high doses, shown some cognitive benefits in Alzheimer’s disease animal models and in aged mice but their effects on cognition in humans, and at tolerable doses, are unknown [15]. 

## 4. Materials and Methods

Details of the design of the ONTRAC trial (Australian New Zealand Clinical Trials Registry number ACTRN12612000625875) have been published previously [5]. Immune-competent, community dwelling adults with at least two histologically confirmed nonmelanoma skin cancers in the past 5 years were randomized to receive nicotinamide 500 mg or placebo twice daily (with or without food) for 12 months. Participants were stratified by gender, study site and by number of nonmelanoma skin cancers in the previous five years (≤5 or ≥6). Participation of ONTRAC patients in the NCF and QoL substudies was not mandatory, and eligibility required spoken and written English language skills equivalent to year 8 to complete the neuropsychological assessments. Assessments were administered at baseline and month 12 by trained research assistants. 

The NCF substudy assessed verbal learning and memory, verbal and written word fluency, information processing, attention/working memory, and executive function using the following battery of instruments: Hopkins Verbal Learning Test-Revised (HVLT-R) [16], Controlled Oral Word Association (COWA) [17], Written Word Fluency [18], Stroop Color and Word Test [19], Trail Making Test Part A and B [20], and the Wechsler Memory Scale-Third edition WMS-III Digit Span [21]. Cognitive symptoms were evaluated using the European Organisation for Research and Treatment of Cancer Quality of life questionnaire (EORTC-QLQ-C30) Cognitive Functioning scale [22].

The QoL substudy used a selection of scales from the Patient Reported Outcomes Measurement Information System Computer Adaptive Test (PROMIS-CAT) system [23] including Global Health (mental and physical), Anxiety, Depression, Fatigue, Applied Cognitive Abilities, and Applied Cognitive General Concerns. 

Raw neuropsychological scores were converted to demographically-corrected T scores (based on age, sex, education, and ethnicity) [24] with an expected mean of 50 and standard deviation of 10. Comparisons between treatment arms at 12 months on the scaled scores from these assessments were performed, as specified a priori in the ONTRAC analysis plan [5], using a linear model with the baseline value, treatment allocation, and centre fitted as covariates. 

## 5. Conclusions

Nicotinamide at a dose of 500 mg twice daily for 12 months did not significantly alter neurocognitive function in our participants. It may be that higher nicotinamide doses than our skin cancer chemoprevention dose, administered over a longer duration, are needed to elicit beneficial effects on NCF.

## Figures and Tables

**Table 1:** Baseline characteristics.

Characteristic	NCF Subgroup *		All ONTRAC Patients	
	Nicotinamide N = 152	Placebo N = 158	Nicotinamide N = 193	Placebo N = 193
Age Mean (SD)	66.3 (11.0)	65.8 (11.7)	66.4 (11.8)	66.4 (11.8)
Female	55 (36.2%)	60 (38.0%)	71 (36.8%)	72 (37.3%)
Years of education (SD)	13.0 (4.0)	12.7 (4.1)	12.5 (4.0)	12.6 (4.4)
Never smoked	72 (47.4%)	75 (47.5%)	92 (47.7%)	88 (45.6%)
NMSCs in previous 5 years Mean (SD)	8.2 (8.7)	8.3 (7.4)	7.9 (8.0)	8.2 (7.4)
Hypertension	68 (44.7%)	70 (44.3%)	86 (44.6%)	84 (43.5%)
Hypercholesterolaemia	63 (41.4%)	60 (38.0%)	79 (40.9%)	82 (42.5%)
Asthma	28 (18.4%)	19 (12.0%)	37 (19.2%)	21 (10.9%)
Ischaemic heart disease	23 (15.1%)	18 (11.4%)	32 (16.6%)	23 (11.9%)
Osteoporosis	11 (7.2%)	15 (9.5%)	19 (9.8%)	20 (10.4%)
Cancer (other than skin)	9 (5.9%)	8 (5.1%)	14 (7.3%)	10 (5.2%)
Diabetes	11 (7.2%)	12 (7.6%)	16 (8.3%)	15 (7.8%)
Stroke/TIA	5 (3.3%)	12 (7.6%)	5 (2.6%)	13 (6.7%)

NCF, neurocognitive function; NMSCs, nonmelanoma skin cancers; TIA, transient ischaemic attack. * Values for patients that contributed both baseline and month 12 data shown.

**Table 2:** NCF and QoL Substudy Results.

Scale	Nicotinamide Mean (SD)	Placebo Mean (SD)	Estimated Effect (95% CIs) ^†^
**NCF Substudy**	N = 152 *	N = 158 *	
**Cognitive Domain**			
Test			
**Verbal Learning and Memory**			
HVLT-R Total recall			
Baseline	39.68 (10.76)	40.06 (11.42)	
Change to Month 12	6.03 (9.64)	4.54 (10.97)	1.22 (−0.79 to 3.23; *p* = 0.23)
HVLT-R Delayed recall			
Baseline	37.41 (12.61)	38.67 (12)	
Change to Month 12	5.5 (10.25)	3.77 (9.47)	1.29 (−0.75 to 3.33; *p* = 0.22)
**Verbal Fluency**			
COWA Total letter fluency			
Baseline	51.45 (12.02)	51.18 (13.39)	
Change to Month 12	2.37 (7.5)	1.85 (8.02)	0.54 (−1.15 to 2.22; *p* = 0.53)
COWA Category fluency—animal			
Baseline	50.86 (10.22)	51.05 (10.29)	
Change to Month 12	0.41 (9.39)	1.3 (10.69)	−1.00 (−2.89 to 0.89; *p* = 0.30)
**Written Fluency**			
Baseline	54.32 (10.92)	52.24 (11.78)	
Change to Month 12	1.63 (6.07)	1.68 (6.18)	0.14 (−1.22 to 1.50; *p* = 0.84)
**Executive Function**			
Trails B			
Baseline	54.37 (9.51)	54.7 (9.06)	
Change to Month 12	1.24 (7.92)	0.8 (7.85)	0.30 (−1.32 to 1.93; *p* = 0.71)
**Stroop Colour-Word**			
Baseline	49.32 (8.5)	50.4 (9.11)	
Change to Month 12	0.15 (5.41)	0.25 (5.15)	−0.34 (−1.48 to 0.80; *p* = 0.56)
Colour-word (inference)			
Baseline	46.29 (7.69)	46.76 (6.92)	
Change to Month 12	0.23 (5.91)	0.11 (5.13)	−0.10 (−1.2 to 1.00; *p* = 0.86)
**Attention**			
Digit span total			
Baseline	56.97 (10.26)	56.1 (10.02)	
Change to Month 12	2.39 (6.54)	0.63 (7.39)	1.90 (0.40 to 3.39; *p* = 0.01)
**Information Processing**			
Trails A			
Baseline	50.81 (8.38)	50.93 (9.69)	
Change to Month 12	1.59 (7.84)	1.52 (8.72)	0.00 (−1.64 to 1.65; *p* = 1.00)
**Symptoms**			
QLQ-C30-Cog. Functioning			
Baseline	76.32 (22.16)	73.99 (17.43)	
Change to Month 12	1.97 (18.79)	1.06 (19.31)	2.03 (−1.42 to 5.50; *p* = 0.25)
**QoL Substudy**	N = 143 *	N = 146 *	
Scale			
Global Mental Health			
Baseline	52.04 (8.41)	51.69 (7.89)	
Change to Month 12	−0.31 (5.73)	−0.38 (6.57)	0.12 (−1.26 to 1.50; *p* = 0.87)
Global Physical Health			
Baseline	52.98 (8.01)	51.31 (7.44)	
Change to Month 12	−1.12 (5.76)	−0.87 (5.63)	0.10 (−1.18 to 1.38; *p* = 0.88)
Anxiety			
Baseline	50.02 (7.96)	50.21 (7.66)	
Change to Month 12	0.12 (6.41)	0.90 (6.88)	−0.81 (−2.25 to 0.62; *p* = 0.27)
Depression			
Baseline	47.51 (8.03)	47.99 (7.11)	
Change to Month 12	0.86 (6.56)	0.80 (6.51)	−0.10 (−1.49 to 1.29; *p* = 0.89)
Fatigue			
Baseline	47.62 (8.57)	49.13 (7.68)	
Change to Month 12	0.33 (7.10)	0.09 (6.90)	−0.28 (−1.77 to 1.21; *p* = 0.71)
Applied Cog Abilities			
Baseline	51.68 (6.69)	50.11 (6.40)	
Change to Month 12	−0.90 (6.53)	0.17 (6.87)	−0.31 (−1.70 to 1.08; *p* = 0.66)
Applied Cog General Concerns			
Baseline	33.81 (9.27)	35.75 (8.19)	
Change to Month 12	−0.45 (8.83)	−0.19 (8.16)	−1.15 (−2.89 to 0.59; *p* = 0.19)

* Values for patients that contributed at least one scale score at both baseline and month 12 data shown. Change in score is the difference between the baseline score and the 12 month score. ^†^ Estimates from a linear model with the baseline value, treatment allocation, and centre fitted as covariates. COWA = Controlled Oral Word Association (of the Multilingual Aphasia Examination); Digit span = Wechsler Memory Scale—Third edition Digit Span; Hopkins Verbal Learning Test-Revised = HVLT-R; Trails A = Trail Making Test Part A; Trails B = Trail Making Test Part B.

## Supplementary Materials

The following are available online at https://www.mdpi.com/2308-3417/4/1/31/s1. Figure S1: CONSORT diagram for neurocognitive function (NCF) testing of ONTRAC participants; Figure S2: CONSORT diagram for Quality of Life (QoL) assessment of ONTRAC participants.
