# Supplementary material for: Neurocognitive Function and Quality of Life Outcomes in the ONTRAC Study for Skin Cancer Chemoprevention by Nicotinamide
Source: Geriatrics (Basel). 2019 Mar 25;4(1):31. doi: 10.3390/geriatrics4010031 (PMC6473406; doi:10.3390/geriatrics4010031)

SUPPLEMENTARY FIGURE S1: CONSORT diagram for neurocognitive function (NCF) testing of ONTRAC participants

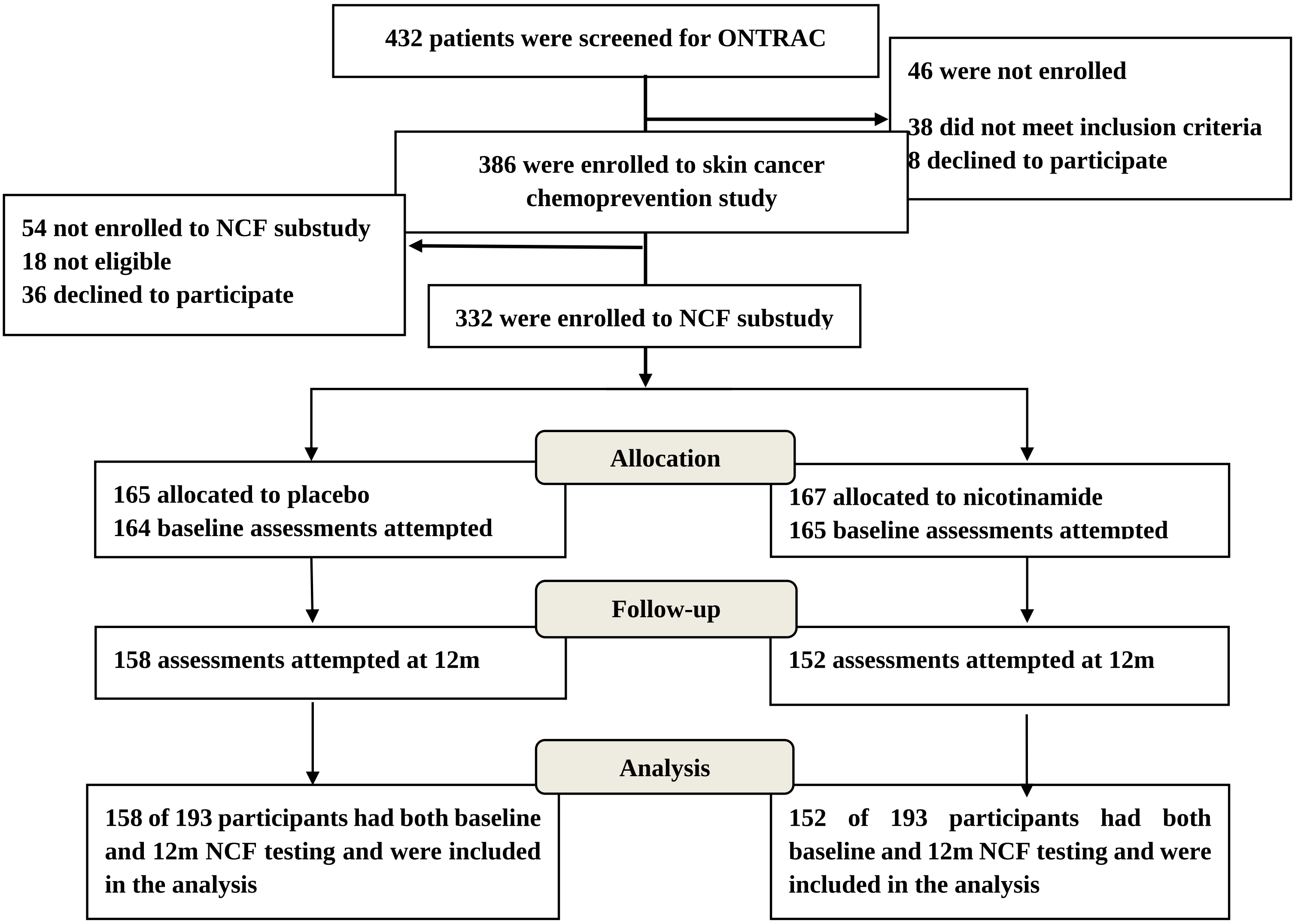

SUPPLEMENTARY FIGURE S2: CONSORT diagram for Quality of Life (QoL) assessment of ONTRAC participants

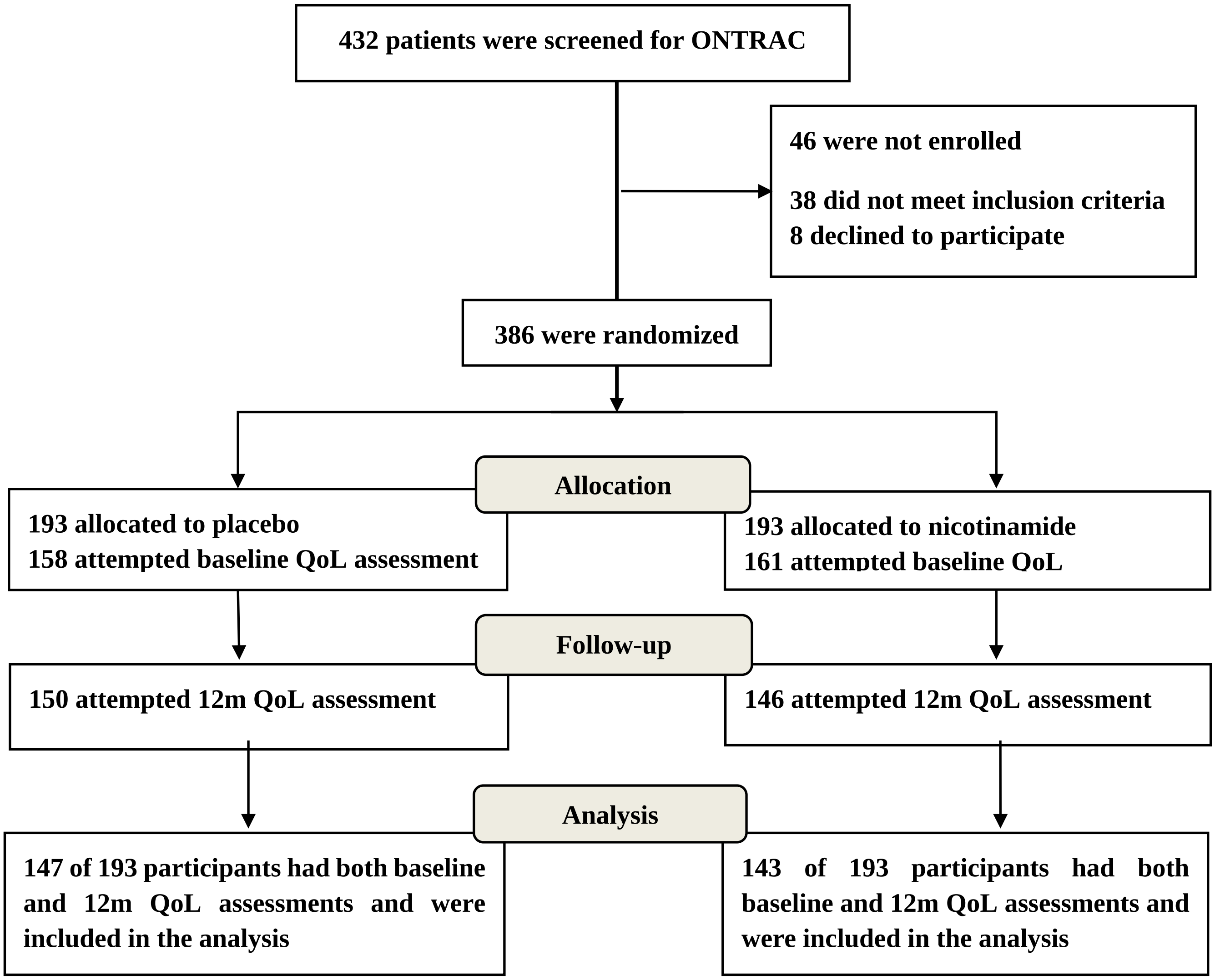

Supplement: Supplementary file 1 [file geriatrics-04-00031-s001.pdf]
